# Exploring the Association Between Colorectal Cancer Metastasis and Metabolic Dysfunction-Associated Steatotic Liver Disease: A Nationwide Analysis

**DOI:** 10.14740/wjon2809

**Published:** 2026-07-08

**Authors:** Mariam Alamgir, Joanne Lin, Anmol Mohan, Carol Singh, Aalam Sohal, Sunny Sandhu, Marina Roytman

**Affiliations:** a School of Medicine, St. George’s University, Grenada, West Indies; b Division of Gastroenterology and Hepatology, University of California, San Francisco, CA, USA; c Department of Internal Medicine, Karachi Medical and Dental College, Karachi, Pakistan; d Department of Internal Medicine, Dayanand Medical College and Hospital, Ludhiana, Punjab, India; e Division of Gastroenterology and Hepatology, Creighton University Phoenix Health Sciences Campus, Phoenix, AZ, USA; f Division of Gastroenterology and Hepatology, Stanford University, Palo Alto, CA, USA; g Division of Gastroenterology and Hepatology, University of California, San Francisco-Fresno, Fresno, CA, USA

**Keywords:** Colorectal cancer, Colorectal cancer metastasis, Metabolic dysfunction-associated steatotic liver disease, Hospital outcomes, National Inpatient Sample

## Abstract

**Background:**

Metabolic syndrome-associated conditions and malignancies frequently co-exist, influencing each other’s progression, severity, and prognosis. This study evaluates whether metabolic dysfunction-associated steatotic liver disease (MASLD) contributes to an increased risk of colorectal cancer (CRC) metastasis and in-hospital mortality using a large nationally representative database.

**Methods:**

We analyzed data from the National Inpatient Sample (NIS) database (2016 to 2020), identifying adult hospitalizations with CRC and then stratified the total population by the presence or absence of MASLD. Outcomes assessed included total CRC metastases, site-specific metastasis (such as gastrointestinal (GI) metastasis, liver metastasis, non-GI metastasis, lymphoid metastasis), and in-hospital mortality. Multivariate logistic regression model was used to adjust for patient demographics, comorbidities, and hospital characteristics. A subgroup analysis was performed to assess the association of MASLD and liver metastasis due to differences in sex, age, and race.

**Results:**

Among 814,270 hospitalizations with CRC, 35,595 (4.4%) had concurrent MASLD diagnosis. Hospitalizations with MASLD had a significantly higher prevalence of total metastasis (47.9% vs. 42.2%), liver metastasis (30.5% vs. 23.8%), GI metastasis (12.8% vs. 11.8%), and lymphoid metastasis (11.9% vs. 10.6%). Multivariate analysis also showed that MASLD was independently associated with higher odds of liver metastasis (adjusted odds ratio (aOR) 1.38, 95% confidence interval (CI) 1.31–1.46), total metastasis (aOR 1.24, 95% CI 1.18–1.30), GI metastasis (aOR 1.08, 95% CI 1.00–1.16), and lymphoid metastasis (aOR 1.12, 95% CI 1.04–1.21). Analysis also revealed an increased odds of in-hospital mortality (aOR 1.85, 95% CI 1.68–2.03) among hospitalizations with MASLD. The association of MASLD with liver metastasis was consistent across sex, age, and race.

**Conclusion:**

MASLD is significantly associated with metastatic CRC, especially liver metastasis, and increased in-hospital mortality. These findings support the need for increased surveillance and effective treatment for concurrent MASLD in patients with CRC.

## Introduction

Metabolic dysfunction-associated steatotic liver disease (MASLD) has an estimated global prevalence of 32% and is now the leading cause of chronic liver disease worldwide [1, 2]. Metabolic dysfunction-associated conditions and malignancy frequently co-exist and can significantly impact the progression and prognosis of one another. MASLD is a well-established risk factor for hepatocellular carcinoma (HCC) [3], and studies show evidence for several other gastrointestinal (GI) cancers such as colorectal, pancreatic, and esophageal cancers [4]. Studies also link MASLD to an increased risk for non-GI cancers including lung, breast, and gynecological cancers [5].

According to GLOBOCAN 2022 data, colorectal cancer (CRC) is the third most commonly diagnosed cancer and the second leading cause of cancer-related deaths worldwide [6]. In 2022, approximately 1.9 million people were diagnosed with CRC, with over 900,000 mortalities [6]. In the United States, an estimated 154,270 new cases of CRC and 52,900 CRC-related deaths are projected for 2025 by the American Cancer Society [7]. The overall 5-year survival rate for early-stage localized CRC is favorable at over 91%, but this drops to 72% for regional disease with lymph node involvement and less than 13% in patients with distant metastases [8]. The liver is the most common site of CRC metastasis, followed by the lungs [9]. Patients with CRC and liver metastasis have a high mortality rate, and recent evidence suggests that the presence of MASLD may increase the risk of CRC metastasis [10, 11].

Prior studies have demonstrated that MASLD contributes to a lipid-rich hepatic microenvironment that supports the growth and metastatic colonization of CRC cells through upregulation of fatty acid synthase (FASN), activation of epidermal growth factor receptor (EGFR) through palmitoylation, and an increase in immunosuppressive cells, such as M2-type tumor-associated macrophages (TAMs) and myeloid-derived suppressor cells (MDSCs), alongside a reduction in anti-tumor immune cells such as natural killer (NK) and cytotoxic T lymphocytes [12, 13]. This altered immunologic environment allows cancer cells to evade immune detection and proliferate within the liver [13]. While *in vitro* studies have highlighted the role of the lipid-rich hepatic environment in facilitating metastasis, real-world data on this association remain limited, particularly at a national level [14]. The objective of this study is to evaluate the association between CRC metastasis and MASLD, using a large, nationally representative dataset.

## Materials and Methods

### Data source

This study is a retrospective secondary analysis of data obtained from the publicly available National Inpatient Sample (NIS) database for the period 2016 to 2020. The NIS is the largest publicly available inpatient database in the United States, managed by the Healthcare Cost and Utilization Project (HCUP) and sponsored by the Agency for Healthcare Research and Quality (AHRQ) [15]. It contains a 20% stratified sample of hospital discharges, offering reliable estimates of disease burden and outcomes. All hospitalizations in the NIS are de-identified and recorded as unique entries.

### Ethical statements

This study used publicly accessible, de-identified data from the NIS database. The research adhered to the ethical guidelines of the responsible institution for human subject studies and complied with the principles outlined in the Declaration of Helsinki. Institutional Review Board (IRB) approval was not necessary as the data is both publicly available and de-identified.

### Study population

Adult hospitalizations (aged > 18 years) with a diagnosis of CRC were identified from the NIS database between 2016 and 2020 using International Classification of Diseases, 10th Revision (ICD-10) codes. Hospitalizations with missing demographic details or with incomplete mortality data were excluded from the analysis. The cohort was then divided into two distinct groups based on the presence or absence of MASLD, determined by ICD-10 codes for nonalcoholic fatty liver disease (NAFLD), the prior nomenclature for MASLD. MASLD was identified using the following approach. ICD-10 codes were used to identify hospitalizations with liver disease without mention of alcohol (ICD-10: I85.xx, K65.2, K72.1x, K72.9x, K74.0-2, K74.6x, K75.8x, K75.9, K76.0, K76.6-9, K77, R18.x). We then excluded hospitalizations with chronic liver disease with mention of alcohol or alcohol use disorder, alcoholic cirrhosis, autoimmune hepatitis, Budd-Chiari syndrome, chronic passive congestion of liver, clonorchiasis, disorders of porphyrin and bilirubin metabolism, echinococcus of liver, fascioliasis, Gaucher disease, hemochromatosis, viral hepatitis, lysosomal acid lipase deficiency and other lipoid disorders, opisthorchiasis, other amyloidosis, other deficiencies of circulating enzymes, other disorders of the liver, primary biliary cholangitis, primary sclerosing cholangitis, syphilis of the liver, or Wilson disease. This approach has been used in previous studies. This has been used in previous studies [16, 17].

### Study variables

Data were obtained on patient demographics, including age, sex, and race, primary insurance, and median income quartile. Information was also obtained on comorbidities such as diabetes, hypertension, chronic pulmonary disease, coronary artery disease (CAD), chronic kidney disease (CKD), congestive heart failure (CHF), coagulopathy, obesity, gastroesophageal reflux disease (GERD), obstructive sleep apnea (OSA), hyperlipidemia, and smoking.

### Study outcomes

The primary outcomes were the prevalence of total CRC metastasis (including site-specific metastases such as liver, GI, non-GI, and lymphoid) among hospitalizations with and without MASLD. GI metastasis refers to the metastasis to GI tract and peritoneum. Lymphoid metastasis refers to secondary malignancy of lymph nodes. Non-GI metastasis refers to metastasis to mediastinum, lung, pleura, kidney, bladder, skin, brain and cerebral meninges, bone marrow, adrenal gland, and ovary. The secondary outcome was in-hospital mortality. Total metastasis represents the presence of any metastatic ICD-10 code. The ICD-10 codes are provided in the Supplementary Material 1 (wjon.elmerpub.com).

### Statistical analysis

Hospital-level discharge weights from the NIS were utilized to produce national estimates and reported in the results section. The study accounted for strata and hospital clustering to obtain valid standard errors and confidence intervals (CIs). Categorical and continuous variables were compared using Chi-square tests and independent sample *t*-tests, respectively. All binary outcomes were analyzed using multivariate logistic regression. Covariates included in the regression model were age, sex, race, primary payer, median household income, and comorbidities (diabetes, hypertension, chronic pulmonary disease, CAD, CKD, GERD, OSA, CHF, coagulopathy, obesity, chronic obstructive pulmonary disease (COPD), and smoking). Since in-hospital mortality can be confounded by metastasis, we performed additional analysis assessing the association between MASLD and in-hospital mortality, by including total metastasis as a covariate. To assess the effect modification, we tested interaction between MASLD and sex, age, and race categories on liver metastasis using survey-weighted logistic regression models, each adjusted for full covariate set. Adjusted odds ratios (aORs) with 95% CIs were calculated. A type I error rate of less than 0.05 was deemed statistically significant. All analyses were performed using STATA version 17.0 [18].

## Results

### Patient characteristics

Of 814,270 hospitalizations with CRC included in the study, 35,595 (4.4%) were associated with a concurrent MASLD diagnosis (Table 1). Hospitalizations with MASLD were younger, with 56.1% being over 65 years old, compared with 60.1% in the non-MASLD group. The sex distribution was similar between the two groups, with 51.2% of hospitalizations in the MASLD group being male, compared with 50.7% in the non-MASLD group. In terms of race, the majority of hospitalizations in both groups were White, with 70.5% in the non-MASLD group and 70.2% in the MASLD group. Other racial groups, such as African American, Hispanic, Asian/Pacific Islander, and Native American, were represented in smaller percentages, with significant differences in distribution between the MASLD and non-MASLD groups.

**Table 1:** Patient Demographics Among Colorectal Cancer-Related Hospitalizations, Stratified by the Presence of MASLD

Demographics	Absence of MASLD, n (%)	Presence of MASLD, n (%)	P-value
Age			< 0.001
< 45 years	45,180 (5.8)	1,850 (5.2)	
45–64 years	265,930 (34.2)	13,775 (38.7)	
> 65 years	467,565 (60.1)	19,970 (56.1)	
Sex			0.371
Males	394,740 (50.7)	18,240 (51.2)	
Females	383,935 (49.3)	17,355 (48.8)	
Race			< 0.001
White	548,785 (70.5)	24,970 (70.2)	
African Americans	109,525 (14.1)	4,185 (11.8)	
Hispanic	70,200 (9.0)	3,995 (11.2)	
Asian/Pacific Islander	25,160 (3.2)	1,225 (3.4)	
Native American	3,470 (0.4)	200 (0.6)	
Other	21,535 (2.8)	1,020 (2.9)	
Primary expected payer			< 0.001
Medicare	457,335 (58.7)	19,640 (55.2)	
Medicaid	76,480 (9.8)	3,735 (10.5)	
Private	208,835 (26.8)	10,470 (29.4)	
Uninsured	17,545 (2.3)	865 (2.4)	
Median household income			0.706
Lowest quartile	221,725 (28.5)	10,140 (28.5)	
Second quartile	205,890 (26.4)	9,200 (25.9)	
Third quartile	186,320 (23.9)	8,600 (24.2)	
Highest quartile	164,740 (21.2)	7,655 (21.5)	

n (%): number of hospitalizations in each group (percentage of hospitalizations). MASLD: metabolic dysfunction-associated steatotic liver disease.

### Comorbidities

Hospitalizations with MASLD had a significantly higher prevalence of several comorbidities (Table 2). Notably, 31.9% of hospitalizations with MASLD had diabetes, compared with 26.1% in the non-MASLD group (P < 0.001). The prevalence of obesity was also higher in the MASLD group (17.7% vs. 13.0%, P < 0.001), as were conditions like coagulopathy (3.5% vs. 1.1%, P < 0.001), GERD (22.4% vs. 20.7%, P < 0.001), and OSA (6.6% vs. 5.2%, P < 0.001). There was no significant difference in the prevalence of hypertension, COPD, CAD, CKD, or CHF between the two groups.

**Table 2:** Comorbidities Among Colorectal Cancer-Related Hospitalizations, Stratified by the Presence of MASLD

Comorbidities	Absence of MASLD, n (%)	Presence of MASLD, n (%)	P-value
Diabetes	203,515 (26.1)	11,340 (31.9)	< 0.001
Hypertension	471,660 (60.6)	21,430 (60.2)	0.539
Chronic pulmonary disease	133,765 (17.2)	6,005 (16.9)	0.504
Coronary artery disease	134,245 (17.2)	5,840 (16.4)	0.077
Chronic kidney disease	112,920 (14.5)	5,440 (15.3)	0.071
Congestive heart failure	105,705 (13.6)	4,640 (13.0)	0.19
Coagulopathy	8,260 (1.1)	1,245 (3.5)	< 0.001
Smoking	274,200 (35.2)	12,445 (34.9)	0.673
Obesity	101,390 (13.0)	6,300 (17.7)	< 0.001
Gastroesophageal reflux disease	161,355 (20.7)	7,985 (22.4)	< 0.001
Obstructive sleep apnea	40,450 (5.2)	2,365 (6.6)	< 0.001
Hyperlipidemia	236,290 (30.4)	10,475 (29.4)	0.112

n (%): number of hospitalizations in each group (percentage of hospitalizations). MASLD: metabolic dysfunction-associated steatotic liver disease.

### Outcomes

**Figure 1:**
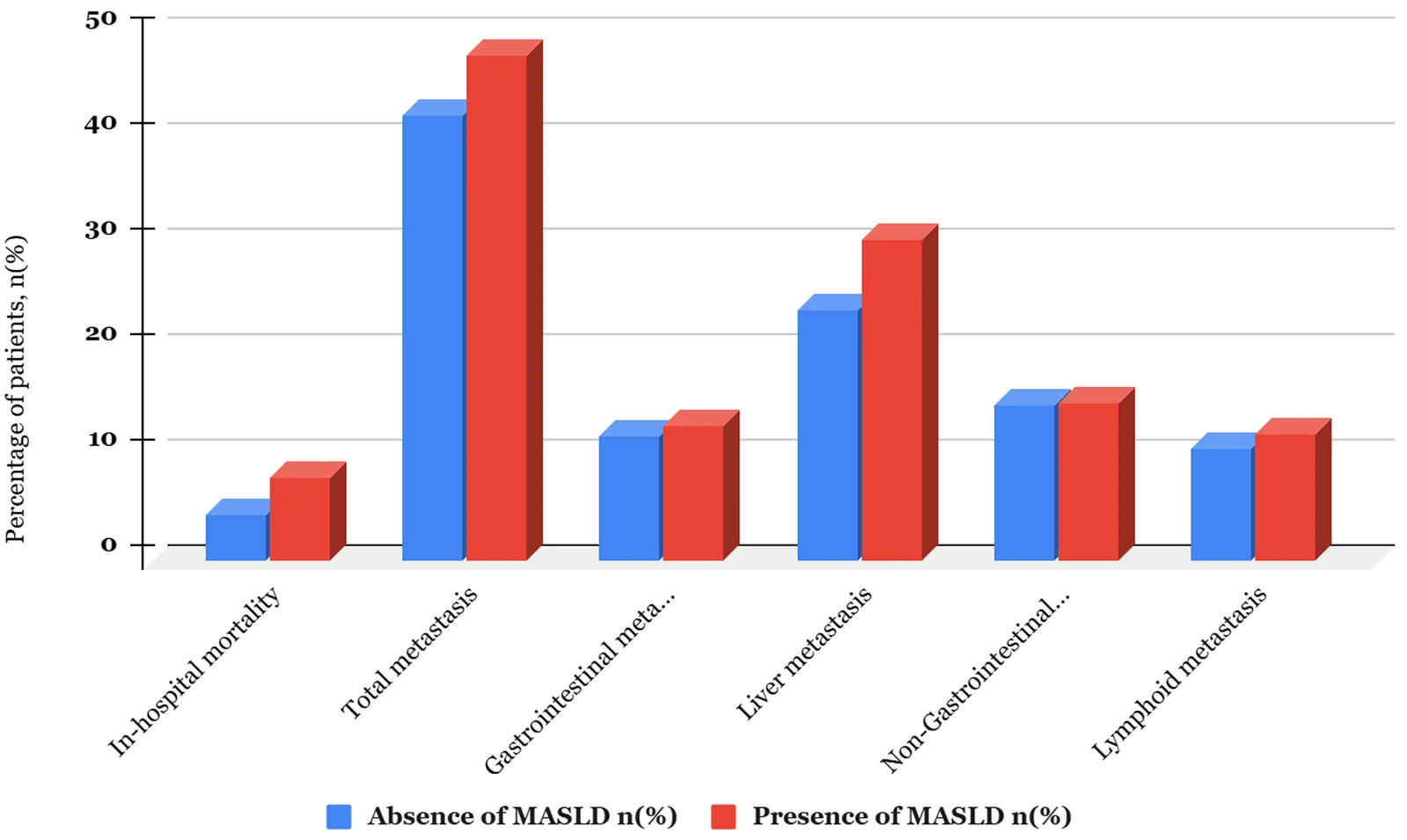
Bar chart comparing outcomes, stratified by the presence of MASLD among colorectal cancer-related hospitalizations. MASLD: metabolic dysfunction-associated steatotic liver disease.

Table 3 and Figure 1 demonstrate the outcomes among CRC hospitalizations, stratified by the presence of MASLD. The results of the multivariate logistic regression model for these outcomes are presented in Table 4.

**Table 3:** Outcomes Among Colorectal Cancer-Related Hospitalizations, Stratified by the Presence of MASLD

Outcomes	Absence of MASLD, n (%)	Presence of MASLD, n (%)	P-value
In-hospital mortality	33,483 (4.3)	2,812 (7.9)	< 0.001
Total metastasis	328,600 (42.2)	17,050 (47.9)	< 0.001
Gastrointestinal metastasis	91,535 (11.8)	4,570 (12.8)	0.006
Liver metastasis	185,160 (23.8)	10,845 (30.5)	< 0.001
Non-gastrointestinal metastasis	114,210 (14.7)	5,330 (14.9)	0.487
Lymphoid metastasis	82,790 (10.6)	4,235 (11.9)	< 0.001

n (%): number of hospitalizations in each group (percentage of hospitalizations). MASLD: metabolic dysfunction-associated steatotic liver disease.

**Table 4:** Multivariate Logistic Model Regression Examining the Association Between MASLD and Outcomes Among Colorectal Cancer-Related Hospitalizations

Outcomes	aOR	95% CI	P-value
In-hospital mortality	1.85	1.68-2.03	< 0.001
Total metastasis	1.24	1.18-1.30	< 0.001
Gastrointestinal metastasis	1.08	1.00-1.16	0.03
Liver metastasis	1.38	1.31-1.46	< 0.001
Non-gastrointestinal metastasis	1.01	0.93-1.08	0.86
Lymphoid metastasis	1.12	1.04-1.21	0.003

aOR: adjusted odds ratio; CI: confidence interval; MASLD: metabolic dysfunction-associated steatotic liver disease.

#### Total metastasis

The prevalence of total metastases was significantly higher in hospitalizations with MASLD than in those without MASLD (47.9% vs. 42.2%, P < 0.001). After adjusting for confounding factors, hospitalizations in the MASLD group had statistically significant higher odds of having total metastases (aOR 1.24, 95% CI 1.18–1.30, P < 0.001).

#### GI metastasis

The prevalence of GI metastases was significantly higher in the hospitalizations with MASLD as compared with hospitalizations without MASLD (12.8% vs. 11.8%, P = 0.006). After adjusting for confounding factors, hospitalizations in the MASLD group had a modest but statistically significant higher odds of having GI metastases (aOR 1.08, 95% CI 1.00–1.16, P = 0.03).

#### Liver metastasis

The prevalence of liver metastases was significantly higher in the hospitalizations with MASLD as compared with hospitalizations without MASLD (30.5% vs. 23.8%, P < 0.001). After adjusting for confounding factors, hospitalizations in the MASLD group had statistically significant higher odds of having liver metastases (aOR 1.38, 95% CI 1.31–1.46, P < 0.001).

#### Non-GI metastasis

There was no significant difference between the prevalence of non-GI metastases between the groups (14.9% vs. 14.7%, P = 0.49). After adjusting for confounding factors, hospitalizations in the MASLD group were found to have no statistically significant association between MASLD and non-GI metastases (aOR 1.01, 95% CI 0.93–1.08, P = 0.86).

#### Lymphoid metastasis

The prevalence of lymphoid metastases was significantly higher in the hospitalizations with MASLD as compared with hospitalizations without MASLD (11.9% vs. 10.6%, P < 0.001). After adjusting for confounding factors, hospitalizations in the MASLD group had statistically significant higher odds of having lymphoid metastases (aOR 1.12, 95% CI 1.04–1.21, P = 0.003).

#### In-hospital mortality

In-hospital mortality was significantly higher in the hospitalizations with MASLD as compared with hospitalizations without MASLD (7.9% vs. 4.3%, P < 0.001). On multivariate analysis, after adjusting for confounding factors, hospitalizations with MASLD had a statistically significant higher odds of in-hospital mortality as compared with those in the non-MASLD group (aOR 1.85, 95% CI 1.68–2.03, P < 0.001). We further adjusted for metastasis as it may confound mortality. After adjustment, MASLD continued to be associated with higher odds of in-hospital mortality (aOR 1.76, 95% CI 1.06–1.94, P < 0.001)

### Subgroup analysis

#### Liver metastasis

A prespecified interaction testing evaluating the association between MASLD and liver metastasis in CRC hospitalizations by sex, age, or race is presented in Table 5. Among males, MASLD was associated with a predicted probability of 33.5% (95% CI 32–35%), compared with 25.2% (95% CI 24.9–25.5%) in those without MASLD. Among females, MASLD was associated with a predicted probability of 26.5% (95% CI 25–27.9%), compared with patients without MASLD 22.3% (95% CI 22–22.7%). A significant interaction (aOR 0.83, 95% CI 0.75–0.93, P = 0.001) was noted based on sex. No significant interaction was identified based on age groups. However, the absolute risk difference associated with MASLD showed a descriptive trend across age groups: +10.1 percentage points in patients aged 18–44, +7.5 percentage points in patients aged 45–64 years, and +5 percentage points in patients aged 65 or older.

**Table 5:** MASLD–Liver Metastasis Interaction by Sex, Age, and Race/Ethnicity in CRC Hospitalizations

Variable	No MASLD	MASLD	Absolute risk difference (%)	Interaction aOR	P-value
Sex					
Male	25.2 (24.9–25.5)	33.5 (32.0–35.0)	8.3	Ref	
Female	22.3 (22.0–22.7)	26.5 (25.0–27.9)	4.2	0.83 (0.75–0.93)	0.001
Age-groups					
18–44	34.0 (33.2–34.9)	44.1 (40.8–47.3)	10.1	Ref	-
45–64	26.8 (26.5–27.2)	34.3 (32.9–35.7)	7.5	1.01 (0.80–1.28)	0.920
65+	20.7 (20.4–21.0)	25.7 (24.4–27.1)	5	0.93 (0.74–1.18)	0.565
Race					
White	22.3 (22.1–22.6)	28.8 (27.5–30.1)	6.5	Ref	-
African American	30.3 (29.6–30.9)	38.2 (34.9–41.4)	7.9	1.01 (0.87–1.19)	0.878
Hispanic	24.6 (23.8–25.4)	30.9 (27.8–33.9)	6.3	0.97 (0.83–1.15)	0.752
Asian	22.5 (21.3–23.6)	29.4 (23.7–35.1)	6.9	1.02 (0.76–1.38)	0.875
Native American	22.1 (18.8–25.3)	11.5 (2.3–20.7)	−10.6	0.32 (0.12–0.82)	0.018
Other	25.6 (24.0–27.2)	30.3 (24.3–36.2)	4.7	0.9 (0.66–1.21)	0.475

Predicted probabilities derived from survey-weighted logistic regression margins. aOR: adjusted odds ratio; CRC: colorectal cancer; MASLD: metabolic dysfunction-associated steatotic liver disease.

No significant interaction was identified between MASLD and race/ethnicity for White, African American, Hispanic, Asian, or other racial groups (P > 0.05) with consistent absolute risk differences ranging from +4.7 to +7.9 percentage points across the groups. An interaction was observed for Native American patients (interaction term aOR 0.32, 95% CI 0.12–0.82, P = 0.018). This finding should be interpreted with caution due to small sample size.

## Discussion

In this nationwide analysis of 814,270 hospitalizations with CRC, we found that MASLD was independently associated with a higher odd of liver metastasis, total metastasis, GI metastasis, lymphoid metastasis, and in-hospital mortality. To our knowledge, our study is among the first and largest real-world analyses exploring the clinical impact of MASLD on CRC metastasis at a national level. Our findings align with existing preclinical research, which suggests that the lipid-rich hepatic microenvironment of MASLD fosters conditions conducive for metastatic CRC cell colonization [19, 20].

The increased prevalence of liver metastases in hospitalizations with MASLD in our study corresponds with mechanistic studies indicating that the upregulation of FASN and changes in immune cell populations, such as elevated M2-type macrophages and myeloid-derived suppressor cells (MDSC), may promote an immunosuppressive environment within the liver to support metastatic cancer cell colonization [21, 22]. Our findings support the existing hypothesis that MASLD may be associated with an increased risk of CRC metastasis, particularly to the liver, and highlight the need for prospective studies to further investigate this relationship.

With regard to GI metastases, we found a modest but statistically significant increased odds of GI metastasis among hospitalizations with MASLD compared with those without. Although the mechanisms underlying this association are not fully understood, systemic inflammation and metabolic changes commonly seen in MASLD may contribute to the increased susceptibility of metastatic spread to the GI tract [23]. Conversely, our study did not identify a significant association between MASLD and non-GI metastases. This may suggest that the influence of MASLD may be more specific to certain organ systems, particularly those anatomically or metabolically linked to the liver. The organ-specific pattern of CRC metastasis risk in MASLD supports the hypothesis of a hepatic and GI microenvironment-driven mechanism [12, 13].

In our subgroup analysis evaluating whether the association between MASLD and liver metastasis in CRC hospitalizations differed by sex, age, or race, a statistically significant association was observed in both sexes, with a greater risk in males. This sex-related difference may be attributable to hormonal influences on hepatic lipid metabolism, variations in visceral adiposity, and differences in inflammatory responses between men and women, as reported in previous studies [24–26].

Our analysis also revealed that CRC hospitalized with MASLD had significantly higher odds of in-hospital mortality compared with those without MASLD. Given the observational nature of this study and the high comorbidity burden in this population, it is not possible to determine whether MASLD was a direct contributor to mortality or an associated bystander finding. These results corroborate prior studies that noted patients with hepatic steatosis to have a significantly higher incidence of colorectal liver metastasis recurrence and worse overall and hepatic recurrence-free survival [27, 28]. This elevated risk may reflect a combination of factors, including a higher burden of comorbidities such as diabetes and obesity and a greater likelihood of extensive hepatic metastases.

### Limitations

Despite the strengths of our study, several limitations warrant acknowledgment. First, its retrospective design and reliance on administrative data from the NIS may introduce inherent biases, including potential misclassification due to the use of ICD-10 codes to identify both CRC and MASLD cases. Second, the NIS dataset lacks critical clinical details and lacks critical oncologic variables such as cancer stage, tumor burden, tumor differentiation, molecular subtype, primary tumor location, recurrence patterns, treatment regimens (e.g., chemotherapy or surgical interventions), and longitudinal follow-up, which could significantly impact metastasis patterns and patient outcomes. Third, while we adjusted for a range of demographic factors and comorbidities, there may still be residual confounding from unmeasured variables, such as lifestyle factors (e.g., diet and physical activity), genetic predispositions, cancer stage, frailty, performance status, treatment, and severity of liver disease which could influence both MASLD and CRC progression. Furthermore, the database does not provide information on hepatic steatosis severity, fibrosis stage, cirrhosis status, liver enzymes, imaging findings, or histology. This limits the interpretation of the MASLD–metastasis association. The ICD-10 code for lymphoid metastases does not differentiate between regional and distant spread, which is another limitation of the study.

The prevalence of MASLD in our cohort (4.4%) is substantially lower than the globally reported prevalence of approximately 32% [1]. This discrepancy likely reflects significant underreporting of MASLD in administrative databases, where ICD-10 coding for MASLD/NAFLD is dependent on active clinical documentation and physician awareness. This underreporting introduces a selection bias, as hospitalizations identified with MASLD in our cohort may represent a subset with more clinically recognized or severe disease, which could lead to an overestimation of the association between MASLD and CRC metastasis. This limitation should be considered when generalizing our findings.

A further limitation relates to temporality. The nature of NIS data and its reliance on ICD-10 codes preclude the determination of temporal relationships between MASLD and CRC metastasis. It is possible that in some cases, hepatic steatosis may have developed secondary to hepatotoxic cancer treatments such as chemotherapy-associated steatohepatitis rather than representing a pre-existing condition driving metastatic risk. This potential reverse causality cannot be excluded in our dataset and represents an important consideration when interpreting the directionality of our findings. Finally, as this study is based on hospitalized patients, the findings may not be fully generalizable to the broader CRC population, particularly those managed in outpatient settings.

### Conclusion

Our findings of higher odds of liver and GI metastasis among MASLD-related hospitalizations may emphasize the need of enhanced risk stratification, with particular attention to liver metastasis surveillance. These findings also advocate for the need for a multidisciplinary approach involving hepatologists, oncologists, and gastroenterologists in managing care for CRC patients with MASLD.

## Supplementary Material

Suppl 1 ICD-10 codes used in the study.

## Data Availability

The data supporting the findings of this study are available from the corresponding author upon reasonable request.
