# Supplementary material for: Exploring the Association Between Colorectal Cancer Metastasis and Metabolic Dysfunction-Associated Steatotic Liver Disease: A Nationwide Analysis
Source: World J Oncol. 2026 Jul 8;17(5):717–24. doi: 10.14740/wjon2809 (PMC13568736; doi:10.14740/wjon2809)
Supplement: Supplementary file 1 [file wjon-17-05-717-s001.docx]

**Suppl 1**. ICD-10 codes used in the study

| **Variable** | **ICD-10 Code** | **Site / Description** |
| --- | --- | --- |
| **GI metastasis** | C78.4 | Secondary malignancy of small intestine |
|  | C78.5 | Secondary malignancy of large intestine & rectum |
|  | C78.6 | Secondary malignancy of retroperitoneum & peritoneum |
|  | C78.7 | Secondary malignancy of liver & intrahepatic bile duct |
|  | C78.8 | Secondary malignancy of other & unspecified digestive organs |
| **Non-GI metastasis** | C78.0 | Secondary malignancy of lung (all: unspecified, right, left) |
|  | C78.1 | Secondary malignancy of mediastinum |
|  | C78.2 | Secondary malignancy of pleura |
|  | C78.3 | Secondary malignancy of other & unspecified respiratory organs |
|  | C79.0 | Secondary malignancy of kidney & renal pelvis |
|  | C79.1 | Secondary malignancy of bladder & other urinary organs |
|  | C79.2 | Secondary malignancy of skin |
|  | C79.3 | Secondary malignancy of brain & cerebral meninges |
|  | C79.5 | Secondary malignancy of bone & bone marrow |
|  | C79.6 | Secondary malignancy of ovary |
|  | C79.7 | Secondary malignancy of adrenal gland |
|  | C79.8 | Secondary malignancy of other specified sites |
| **Lymph node metastasis** | C77.x | Secondary malignancy of lymph nodes (all subcodes: axillary, inguinal, intrathoracic, intra-abdominal, head/face/neck, intrapelvic, other, unspecified) |
| **Liver metastasis** | C78.7 | Secondary malignancy of liver & intrahepatic bile duct |
| Diabetes | ynel10, ynel11 | Elixhauser ICD-10 codes for diabetes ynel10 and ynel11 |
| Hypertension | ynel16, ynel31 | Elixhauser ICD-10 codes for diabetes ynel16 and ynel31 |
| Coronary artery disease | I25 | I25 |
| Chronic Kidney Disease | N18 | N18 |
| Congestive Heart Failure | ynch2 | Charlson ICD-10 code for congestive heart failure ynch2 |
| Smoking | F17, Z720, Z87.891 | Codes for tobacco use |
| Obesity | ynel22 | Elixhauser ICD-10 codes for obesity ynel22 |
| MASLD |  | Described in the methods section. |
